# Salt-Assisted Air-Purification of Detonation Nanodiamonds

**DOI:** 10.3390/ma19091832

**Published:** 2026-04-29

**Authors:** Jingyao Deng, Wenjing Ba, Xiaoyu Bi, Houjin Huang

**Affiliations:** Faculty of Chemical Engineering and Energy Technology, Shanghai Institute of Technology, No. 100 Haiquan Road, Shanghai 201418, China

**Keywords:** detonation-synthesized nanodiamonds, salt catalysis, purification

## Abstract

**Highlights:**

- Salt-assisted route enables selective oxidation of sp^2^ carbon in DNDs.
- Alkali cations catalyze sp^2^ carbon oxidation via oxygen spillover–electron transfer.
- Wet-wrapping with KHCO^3^ reduces aggregation and improves DND dispersibility.

**Abstract:**

The widespread application of detonation nanodiamonds (DNDs) is limited by surface-coated non-diamond sp^2^ carbon impurities. In this work, an efficient salt-assisted catalytic purification strategy is developed to achieve selective oxidation removal of sp^2^ carbon. DND black powder was mixed with various chloride, carbonate, and bicarbonate salts and thermally treated in air to systematically investigate the effects of anions and cations on purification efficiency. Thermogravimetric analysis reveals that all tested salts significantly reduce the oxidation onset temperature of sp^2^ carbon and exhibit distinct catalytic trends: for anions, bicarbonates > carbonates > chlorides; for cations, Cs^+^ ≈ K^+^ > Na^+^. Among them, KHCO_3_ introduced via a wet-wrapping method shows the optimal performance, lowering the oxidation temperature by approximately 160 °C. Moreover, the wet-wrapping process effectively suppresses particle sintering and agglomeration during purification, resulting in purified DNDs with reduced average particle size and markedly improved dispersibility. Mechanistic investigations demonstrate that free alkali metal cations act as active sites, preferentially catalyzing sp^2^ carbon oxidation through a synergistic oxygen spillover–electron transfer mechanism. This study provides an effective and highly selective approach for DND purification. The proposed salt-assisted strategy, integrating catalytic oxidation and dispersion control, also offers valuable insights for the preparation of high-performance nanomaterials.

## 1. Introduction

Nanodiamonds (NDs) are a carbon-based nanomaterial with a history spanning over fifty years, first synthesized in the former Soviet Union [1]. Nanodiamonds have garnered significant attention due to their unique properties, such as distinctive fluorescence, large specific surface area, stable chemical characteristics, and excellent biocompatibility [2,3]. Currently, researchers have developed multiple techniques for synthesizing NDs, including the detonation method [4], chemical vapor deposition (CVD) [5,6,7,8], and the high-pressure high-temperature (HPHT) method [4]. In industrial production, detonation is the primary method for manufacturing NDs. Within stainless steel tanks, explosives such as 2,4,6-trinitrotoluene (TNT), cyclotrimethylenetrinitramine (RDX), and trinitrotoluene (TATB) are mixed and detonated under negative oxygen balance conditions [9]. The resulting nanodiamonds typically exhibit small dimensions, with particle sizes ranging from 1 to 10 nm [10]. However, the full realization of NDs’ potential hinges on the efficiency of their production and purification processes, which directly influence key properties such as particle size, impurity content, and functional groups [11,12]. Several methods have been developed to purify NDs by removing surface impurities, including acid treatment [13,14,15], annealing [16], and plasma treatment [17,18]. However, these purification methods have certain limitations: they may reduce the monodispersity of NDs, involve complex operations, cause environmental pollution, and pose safety hazards.

Therefore, attempts have been made to remove most sp^2^ carbon impurities from detonation-synthesized nanodiamonds (DNDs) through oxidation in gases such as air or oxygen, thereby achieving purification [19,20,21]. Petrov et al. [22] subjected DND to high-temperature heating in oxygen-enriched or ozone gas. During the reaction, the non-diamond carbon (primarily graphite) within the DND reacted with ozone, converting into carbon dioxide or carbon monoxide, thereby achieving purification of the DND. Moreover, ozone can etch away non-diamond carbon from the DND surface while introducing oxygen-containing functional groups, thereby achieving modification of the DND. Pichot et al. [23] removed sp^2^ carbon (such as graphite and amorphous carbon) by performing thermal oxidation at 420 °C after acid treatment to eliminate metal impurities following detonation. Experimental results demonstrate that this method can effectively purify detonation soot, yielding nano-diamond powder with a purity approaching 100%. Osswald et al. [24] found that within the temperature range of 375–450 °C, the oxidation rates of sp^2^ and sp^3^ carbon species differ. Within the narrow temperature range of 400–430 °C, oxidation of sp^2^-bonded carbon can occur with little or no diamond loss.

Due to the high temperatures involved in calcination, which poses safety risks and is time-consuming, Zhang et al. [25] drew inspiration from the traditional Chinese snack “sugar-roasted chestnuts” to develop a salt-assisted air oxidation (SAAO) method. SAAO mixes DND with salt crystals prior to oxidation, which accelerates the oxidation process and aids in the removal of impurities. This technology has demonstrated highly efficient purification capabilities, particularly for removing random impurities on NDs, such as ultrafine NDs and non-diamond carbon. Subsequently, the Savinov team [26] built upon this approach by employing isothermal crystallization using saturated salt solutions to coat the NDs with salt, ensuring more uniform salt encapsulation. Moreover, it was demonstrated that the NV^−^/NV ratio of ND treated by this salt coating air oxidation (SCAO) method reached as high as 92%.

In this study, we aim to systematically investigate the catalytic effects of salts formed by different anion-cation combinations (Cl^−^, CO_3_^2−^, HCO_3_^−^; Na^+^, K^+^, Cs^+^) on the high-temperature air purification process of detonation-derived nanodiamond (DND) through a systematic experimental design. The study will precisely evaluate the extent to which various salt additives reduce the oxidation temperature of non-diamond carbon impurities. More importantly, this paper will transcend the apparent catalytic phenomena to reveal their underlying catalytic mechanisms: by employing characterization techniques such as thermogravimetric analysis (TGA) and transmission electron microscopy (TEM), and drawing upon advanced theories from the field of carbon soot-catalyzed combustion, we will construct a catalytic reaction model applicable to the “salt-DND” system. This model will elucidate the pivotal role of free alkali metal cations and their specific pathways of action. Ultimately, through comprehensive evaluation of the crystal structure, surface chemical state, and dispersibility of the purified product, this study will provide robust experimental evidence and theoretical support for developing new, highly efficient, low-temperature, and low-energy-consumption purification processes for nanodiamonds. This advancement will propel the preparation and application of high-performance nanodiamond materials.

## 2. Materials and Methods

### 2.1. DND and Salt Dry-Mixing Method

Add DND (Detonation-Synthesized Nanodiamond, Chengdu Brilliant Pharmaceutical, Chengdu, China) and salt (Shanghai Titan, Shanghai, China) to the grinding mill in a 1:3 mass ratio. Start the mill for 10 s, then turn off the power. After standing for 30 min, remove the mixture to obtain the dry-mixed DND and salt sample.

### 2.2. DND and Salt Wet-Wrapping Method

Add DND and salt to a beaker in a 1:3 mass ratio. Add an appropriate amount of water. Place the beaker in a cell disruptor and pulverize intermittently for 2 h and 30 min, carefully controlling the intervals to prevent water boiling. Remove the beaker and place it in an oven at 80 °C overnight to remove moisture. After removal, transfer the sample to a grinding mill, pulverize it, and mix thoroughly to obtain the wet-wrapped DND and salt sample.

### 2.3. Purification Methods

The calcined samples were first subjected to multiple ultrasonic washes with ultrapure water to thoroughly dissolve and elute the soluble salts from the mixture. The washed samples are then purified by dialysis in a dialysis bag with a molecular weight cut-off of 3500 Da for 72 h, with fresh deionized water replaced every 6 h. This prolonged dialysis effectively removes residual soluble salts from the system, yielding the purified samples.

### 2.4. Characterization

Thermogravimetric Analysis. Thermogravimetric Analysis (TGA) was performed using a STA 409 PC Luxx simultaneous thermal analyzer (Netzsch, Selb, Germany) coupled with a capillary column and QMS 403 a′eolos quadrupole mass spectrometer (Netzsch, Selb, Germany). The temperature range was 30 °C to 600 °C, with a sample heating rate of 5 °C min^−1^.

Dynamic light scattering measurements. Dynamic light scattering (DLS, Zetasizer Nano ZS, Malvern Instruments, Ltd., Malvern, UK) was employed to determine the particle size distribution of nanodiamond dispersions. All test samples had a concentration of 0.5 mg/mL and were dissolved in deionized water; prior to testing, they were sonicated for 30 min.

X-ray Photoelectron Spectrometer. X-ray photoelectron spectroscopy (XPS) analysis was carried out using a K-Alpha X-ray photoelectron spectrometer (Thermo Fisher Scientific, Waltham, MA, USA). A monochromatic Al Kα X-ray source (1486.6 eV) was employed at an operating voltage of 12 kV and a filament current of 12.5 mA, corresponding to a total power of 150 W. Survey spectra were recorded at a pass energy of 150 eV with a step size of 1 eV, while high-resolution spectra were acquired at a pass energy of 20 eV with a step size of 0.05 eV. The base pressure of the analysis chamber was better than 5 × 10^−9^ mbar. All binding energies were calibrated against the adventitious carbon C 1s peak at 284.8 eV.

Transmission electron microscopy. Transmission electron microscopy (TEM) analysis was performed using a Philips Tecnai F30 field emission transmission electron microscope (FEI Company, Hillsboro, OR, USA) at an operating voltage of 200 kV. TEM samples were prepared by dispensing ND colloidal aqueous solution onto carbon-coated Cu grids, followed by drying in ambient air.

X-ray Powder diffractometer. The X-ray Powder diffractometer (XRD) model is the Bruker D8 Advance from Bruker, Bremen, Germany. The selected target material is Cu K-ray with a wavelength of 1.5418 Å. The operating voltage is 40 kV, and the operating current is 40 mA.

## 3. Results

### 3.1. Thermogravimetric Analysis (TGA): Direct Evidence of Salt Catalytic Effects

Thermogravimetric analysis (TGA) is the most critical experimental method for directly evaluating the catalytic effect of salt additives on the oxidative decomposition of non-diamond carbon components. In this study, we conducted TGA tests on all samples under air atmosphere. By analyzing their weight loss behavior, we quantitatively revealed the significant effects and patterns of different salts in lowering the oxidation temperature of sp^2^ carbon.

#### 3.1.1. Oxidation Behavior of Original DND

**Figure 1:**
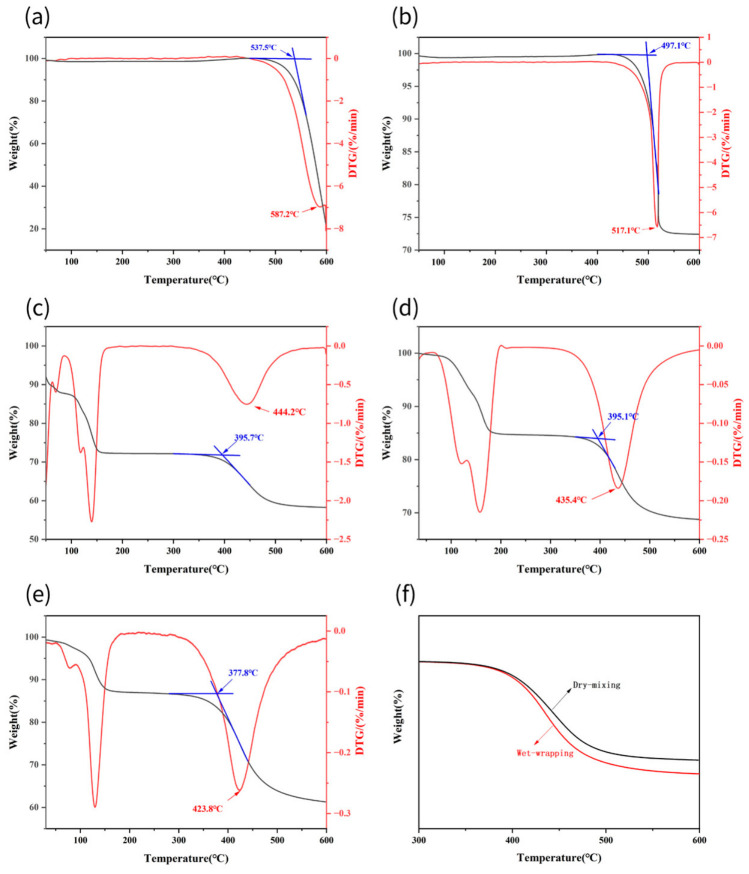
(**a**) TGA of DND; (**b**) and (**c**) TGA of KCl, K_2_CO_3_, and DND mixed in a 3:1 ratio by dry blending; (**d**,**e**) TGA of K_2_CO_3_, KHCO_3_, and DND mixed in a 3:1 ratio by wet-wrapping; (**f**) Localized TGA of K_2_CO_3_ and DND mixed in a 3:1 ratio by dry-mixing and wet-wrapping.

In contrast, the TGA curve of DND exhibits typical non-catalytic oxidation characteristics (Figure 1a). The thermal decomposition process primarily occurs in two stages: First, in the low-temperature region (room temperature ~400 °C), a slight mass loss (approximately 2–5%) is observed. This is mainly attributed to the decomposition of adsorbed water, residual solvents, and some unstable surface functional groups. The second stage is the intense oxidation zone (400–650 °C), where the sample undergoes rapid mass loss. The onset oxidation temperature (T_onset_, defined as the initial weight loss temperature extrapolated from TGA) is approximately 537.5 °C. Subsequently, the oxidation rate increased rapidly and peaked at approximately 587.2 °C, corresponding to the maximum weight loss rate temperature (T_peak_) at the peak of the DTG curve. The substantial mass loss during this stage (approximately 70–80% total weight loss) corresponds to the oxidation of sp^2^-hybridized non-diamond carbon (amorphous carbon, graphene shells, etc.) and partial nanodiamonds by atmospheric oxygen into CO/CO_2_.

#### 3.1.2. Revealing the Anionic Effect

Following the introduction of salt additives, the TGA curves of all samples exhibited a significant shift toward lower temperatures, though the extent of this shift varied depending on the type of anion in the salt (Figure 1, using potassium salt as an example).

The chloride salt (KCl) effect. After mixing with KCl, the oxidation process of the sample was significantly accelerated (Figure 1b). T_onset_ decreased to approximately 497 °C, while T_peak_ shifted to approximately 517.1 °C. This indicates that even chemically inert Cl^−^ ions can provide significant catalytic effects when mixed with DND. We attribute this primarily to the “microreactor” effect of molten salts [25]. The melting point of KCl is 770 °C, but at the nanoscale interface, a local liquid phase or eutectic mixture may form at temperatures significantly below its macroscopic melting point [27,28]. This molten environment significantly enhances the mass transfer efficiency of oxygen to the carbon surface, transforming the reaction from a gas–solid two-phase reaction into a more efficient gas–liquid-solid three-phase reaction, thereby reducing the activation energy required for the oxidation reaction [29].

The carbonate (K_2_CO_3_) effect. Samples mixed with K_2_CO_3_ exhibited superior catalytic performance, with T_onset_ and T_peak_ further decreasing to approximately 395.7 °C and 444.2 °C. The catalytic effect of carbonates surpasses that of chlorides, and the reason may extend beyond simple physical mass transfer effects. The CO_3_^2−^ ions preferentially adsorb onto defect or edge sites on the sp^2^ carbon surface. This adsorption effectively polarizes the adjacent C–C bonds and acts as an efficient electron transfer bridge, facilitating electron flow from the carbon skeleton to oxygen molecules (O_2_) adsorbed at K^+^ sites [30]. This process significantly enhances the efficiency of oxygen activation into superoxide species (O_2_^−^), thereby lowering the energy barrier for the initial oxidation reaction. This amplifies the “microreactor” effect of the molten salt, leading to enhanced catalytic performance (Figure 1c).

Bicarbonate (KHCO_3_) effect. Among all tested salts, samples mixed with KHCO_3_ via wet wrapping exhibited the most remarkable catalytic activity. Its oxidation reaction initiates extremely early, with T_onset_ as low as approximately 377.8 °C, while T_peak_ drops significantly to about 423.8 °C (Figure 1e). Its outstanding performance stems from a triple synergistic mechanism. The first is the ultimate blending and contact. The wet wrapping achieved by the pulverizer creates a maximized salt/carbon contact interface, pushing the “microreactor” effect to its fullest potential. Secondly, active species are generated in situ. KHCO_3_ decomposes during heating at temperatures between 100 and 200 °C (2KHCO_3_ → K_2_CO_3_ + H_2_O↑ + CO_2_↑). The released H_2_O and CO_2_ gases exert a physical stripping and perforating effect on the amorphous carbon layer enveloping the diamond, compromising the integrity of the carbon layer and creating pathways for oxygen penetration. Finally, gas-phase catalysis occurs, where the H_2_O and CO_2_ produced by decomposition can themselves act as weak oxidizing agents in the reaction (C + H_2_O → CO + H_2_; C + CO_2_ → 2CO). These reactions typically proceed at lower temperatures [31], further promoting carbon consumption.

#### 3.1.3. Revealing the Cation Effect

**Figure 2:**
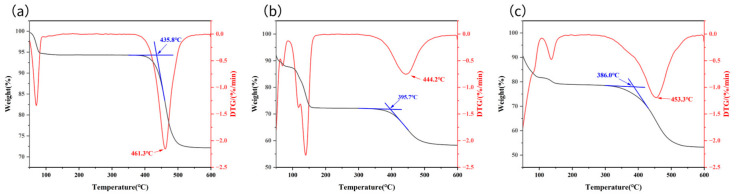
(**a**–**c**) show the TGA curves of Na_2_CO_3_, K_2_CO_3_, Cs_2_CO_3_, and DND dry-mixing at a 3:1 ratio, respectively.

For fixed anions, cations also exhibit a systematic influence on catalytic activity (Figure 2, using the carbonate series as an example).

For the carbonate series, the catalytic activity order is: Cs_2_CO_3_ ≈ K_2_CO_3_ > Na_2_CO_3_. The T_peak_ values for Cs_2_CO_3_ and K_2_CO_3_ samples are very close (approximately 390 °C) and both significantly lower than that of the Na_2_CO_3_ sample (approximately 435.8 °C). A similar trend is observed in the chloride series (CsCl ≈ KCl > NaCl).

This phenomenon can be reasonably explained from the perspectives of the Hofmeister series (ion ordering series) and lattice energy theory [32]. Cs^+^ and K^+^ are classified as “soft” ions, characterized by larger ionic radii and lower charge density [33,34]. The lattice energy formed with anions is relatively low, resulting in corresponding salts typically exhibiting lower melting points. A lower melting point means that during heat treatment, Cs^+^ and K^+^ salts can form a molten state at lower temperatures, enabling the “microreactor” effect to initiate more rapidly and thereby exhibit enhanced catalytic activity. Once in the molten state, the ionic characteristics described by the Hofmeister series begin to dominate their catalytic behavior [35]. Cs^+^ and K^+^, as typical dissociated ions, form a highly dynamic and structurally disordered molten microenvironment. This not only facilitates mass transfer and activation of reactants but also effectively lowers the reaction energy barrier, thereby exhibiting enhanced catalytic activity [36]. In contrast, Na^+^ salts possess higher melting points, resulting in a higher temperature threshold for forming effective liquid-phase “microreactors”. Additionally, Na^+^ exhibits a stronger affinity for liquid ions, potentially forming a relatively ordered and viscous molten environment that hinders efficient reaction progress. Consequently, its catalytic activity is comparatively weaker.

In summary, TGA data provides irrefutable evidence (Table 1) that by selecting appropriate salt types and mixing methods, the oxidation and removal of non-diamond carbon from detonation black powder can be initiated and efficiently completed at temperatures as low as 300–350 °C. This lays a solid foundation for subsequent low-damage, energy-saving purification processes.

### 3.2. Whiteness Analysis: Macro Evaluation of Purification Effectiveness

**Figure 3:**
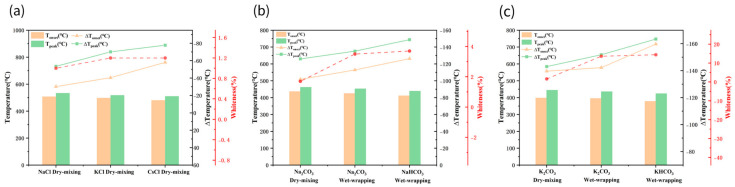
Oxidation temperature data for salt-DND mixtures prepared via different blending methods (**a**–**c**). The red dotted line and coordinate axes indicate the whiteness values of the corresponding groups after calcination at 350 °C for 2 h.

To evaluate the purification effect of different salt additives on detonation-derived nanodiamonds (DNDs) at a macro scale, this study subjected samples to a 2 h isothermal calcination treatment at a fixed temperature of 350 °C and systematically measured the whiteness values of the products. Higher whiteness values indicate whiter samples with fewer sp^2^ heteroatoms. As shown in Table 2, the whiteness value of the raw DND black powder is only 0.8 ± 0.04%, exhibiting a typical deep black powder morphology, while the whiteness after calcination is 0.9 ± 0.03%, with virtually no reduction in sp^2^ carbon content. The whiteness values of DND samples mixed with salt generally align with the trends observed in the measured TGA data (as shown in Figure 3). Notably, the KHCO_3_ and K_2_CO_3_ groups treated via the wet-wrapping method exhibited significantly higher whiteness values than other groups, indicating their superior catalytic performance.

At the same time, we determined the product yields for all experimental groups under uniform calcination conditions of 350 °C for 2 h; the results are shown in Table 2. As a blank control, the yield of the original DND, without any salt added, after calcination under the same conditions was 98.7 ± 0.5%, indicating that without salt catalysis, almost no significant carbon oxidation reaction occurs at 350 °C. The yields of all experimental groups with added salts decreased to varying degrees, and the yields showed a strict negative correlation with whiteness values: the higher the whiteness (the more thorough the removal of sp^2^ carbon), the lower the product yield.

To further evaluate the regulatory role of salt in the oxidation process, additional experiments were conducted under salt-free conditions with calcination at 425 °C for 2 h (Table 2). Under these conditions, the sample exhibited a whiteness of 1.2 ± 0.06 and a yield of 92.8 ± 1.7%, indicating that conventional high-temperature oxidation can only achieve limited removal of sp^2^ carbon and is accompanied by a certain degree of mass loss. In contrast, the system with salt addition achieved a significantly higher increase in whiteness at a lower temperature (350 °C), suggesting that the oxidation effects achievable only at high temperatures in the salt-free system can be efficiently realized at lower temperatures under salt-assisted conditions, with superior purification results. Further analysis of the structural and morphological characterization results presented below confirms that this low-temperature salt-assisted oxidation process did not introduce additional structural defects or result in significant loss of the nanodiamond matrix, demonstrating good reaction selectivity and structural retention capability.

It should be noted that, for the DND purification process, a higher product yield is not necessarily better. A certain degree of quality loss is an inevitable consequence of the selective oxidation and removal of sp^2^ non-diamond carbon impurities, and it is also one of the core objectives of the purification process [24]. An excessively high yield typically indicates that sp^2^ carbon impurities have not been completely removed, preventing the formation of a high-purity diamond phase; conversely, an excessively low yield suggests poor reaction selectivity and unnecessary oxidative loss of the diamond matrix. The optimal KHCO_3_ wet-wrapping system identified in this study significantly increased the whiteness value from the initial 0.8 ± 0.04 to 14.4 ± 0.11 while maintaining a high product yield of 83.8 ± 3.6%, thereby achieving an optimal balance between purification efficiency and product yield. This demonstrates that the method exhibits excellent catalytic selectivity.

### 3.3. Effect of Salt Mixing Method on the Dispersibility of DND Carbon Black

**Figure 4:**
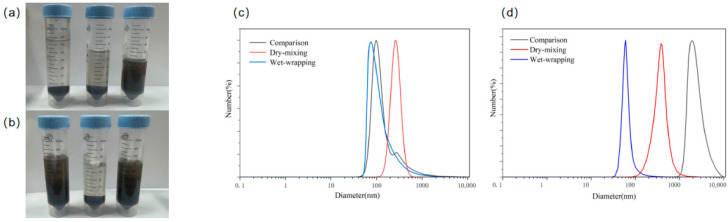
(**a**,**b**) show DND suspensions obtained after mixed calcination treatment using different methods in the Na_2_CO_3_ and K_2_CO_3_ systems, respectively. The leftmost sample represents the control group, the middle sample represents the dry mixing group, and the right sample represents the wet coating group. (**c**,**d**) show the particle size distribution curves of samples obtained after mixed calcination treatment using different methods in the Na_2_CO_3_ and K_2_CO_3_ systems, respectively.

To investigate the effect of different salt mixing methods on the structure of the reactant interface, we compared the macroscopic state and microscopic dispersion of samples after dry mixing and wet-wrapping mixing catalysis. Among these, samples treated with sodium carbonate underwent calcination at 350 °C for 120 min, while samples treated with potassium carbonate underwent calcination at 325 °C for 120 min. To eliminate the dispersing effect of the pulverizer itself on DND black powder, a control group was added. The control group consisted of DND carbon black powder first dispersed using a pulverizer, followed by dry mixing with salt. All samples obtained were washed with water and dialyzed to remove the corresponding salts. As shown in Figure 3a, the product treated by the carbonate dry-mixing process still exhibits significant phase separation, with DND particles agglomerating into larger aggregates due to strong van der Waals forces. In stark contrast, samples treated with wet coating via a pulverizer formed relatively stable suspensions (Figure 4a,b), indicating that wet wrapping significantly improved the dispersion of DND.

To systematically evaluate the effect of salt-assisted calcination on the dispersion properties of detonation-synthesized nanodiamonds, dynamic light scattering (DLS) was used to comprehensively characterize samples treated with different salt systems and mixing methods. Four particle size parameters—Z-average hydrodynamic diameter, intensity mean, volume mean, and number mean diameters—as well as the polydispersity index (PDI) and zeta potential were measured simultaneously. The results are summarized in Table 3 and the corresponding particle size distribution curves are shown in Figure 4. The combination of multidimensional particle size data provides a more accurate reflection of the particle agglomeration state: the intensity mean is most sensitive to large agglomerates, the volume mean reflects the mass fraction of particles of different sizes, and the number mean reflects the true size of monodisperse particles.

The crude, unpurified DND sample exhibited typical severe agglomeration, with a Z-average particle size of 724.67 ± 8.56 nm and a PDI as high as 0.57 ± 0.05, indicating the presence of a large number of micron-sized hard agglomerates in the system; its zeta potential was +37.26 ± 0.67 mV, with the positive charge originating from protonated nitrogen-containing functional groups introduced to the surface during the detonation process. Although the raw particles exhibit some electrostatic repulsion, van der Waals forces increase exponentially with particle size, ultimately leading to highly prone sedimentation.

In the Na_2_CO_3_ system, the wet wrapping–calcination process demonstrated the most effective improvement in dispersion: the Z-average particle size of the sample decreased significantly to 199.0 ± 5.25 nm, the PDI dropped to 0.23 ± 0, and the number-average particle size decreased significantly, indicating that large agglomerates were effectively disaggregated. In contrast, dry mixing could only break up some of the soft agglomerates; the number-average particle size remained large with significant error, suggesting that mechanical mixing alone cannot create a uniform salt-isolated environment during calcination, and secondary agglomeration still occurs at high temperatures. The control group treated only by ultrasonic dispersion had particle sizes intermediate between the wet and dry methods, but the error in the volume-average particle size was extremely large, indicating that a small amount of unbroken large agglomerates remained in the system.

Similar trends were further confirmed in the K_2_CO_3_ system. The number mean particle size of the samples obtained via wet wrapping followed by calcination was the smallest among all experimental groups, with the most thorough decumulation of agglomerates; the PDI was only 0.25 ± 0.4, indicating excellent monodispersity. In contrast, the dispersion effect of the dry-mixed samples was significantly weaker than that of the wet-mixed samples. Notably, the control group—which underwent ultrasonication followed by dry wrapping and calcination—exhibited exceptionally severe high-temperature agglomeration. All particle size parameters surged to the micron range with closely clustered values, and the PDI reached as high as 0.45 ± 0.16. This clearly demonstrates that if the salt and DND are not thoroughly mixed, effective spatial separation cannot be achieved, leading to irreversible sintering and agglomeration of the particles at high temperatures, which in turn causes a drastic deterioration in dispersion.

For all samples subjected to calcination and oxidation, the zeta potential became negative, and the absolute value of the negative potential showed a strong positive correlation with the degree of dispersion improvement. This phenomenon can be explained by changes in the surface chemical structure: high-temperature calcination removes protonated nitrogen-containing cationic groups from the surface while selectively removing the sp^2^ carbon layer. The exposed diamond surface is then oxidized, introducing a large number of acidic oxygen-containing functional groups such as carboxyl and lactone groups, thereby enhancing electrostatic repulsion between particles. Among these, the K_2_CO_3_ wet-wrapping system, which exhibits the highest catalytic activity, achieves a zeta potential of −28.00 ± 1.05 mV, thereby simultaneously optimizing both purification and dispersion.

This significant improvement in dispersion is crucial for subsequent catalytic purification processes. As described in Section 3.4, the catalytic effect of salts depends on their close contact with carbon impurities to form “microreactors”. The extensive and uniform contact interface created by the wet-wrapping ensures that molten salts can maximally envelop each minute carbon particle during heat treatment, thereby maximizing the “microreactor” effect. This provides crucial physical structural evidence explaining why the carbonate system exhibits optimal catalytic activity (as demonstrated by the TGA data in Section 3.1): namely, the wet-wrapping mixture creates prerequisites for efficient catalysis by optimizing the spatial distribution of reactants.

### 3.4. Structural Characterization of Purified Product: Validation of Low-Temperature Purification Efficacy

Thermogravimetric analysis (TGA) thermodynamically and kinetically predicts the feasibility of salt-assisted low-temperature purification. However, the efficacy of this strategy and its core advantage—maximizing retention of nanodiamonds while efficiently removing impurities—must be confirmed through direct structural characterization of the purified product. This section employs X-ray photoelectron spectroscopy (XPS), transmission electron microscopy (TEM) and X-ray diffraction (XRD) to comprehensively characterize representative samples, providing the most direct and compelling evidence for the success of the low-temperature catalytic purification strategy. Specifically, XPS quantitatively analyzes the surface chemical composition and the relative content ratio of sp^3^/sp^2^ hybridized carbon, directly verifying the selective removal of sp^2^ non-diamond carbon impurities; TEM and XRD are further used to examine the morphology, particle size and crystal structure of the samples, confirming the integrity of the diamond phase during the purification process.

#### 3.4.1. X-Ray Photoelectron Spectroscopy (XPS) Analysis

**Figure 5:**
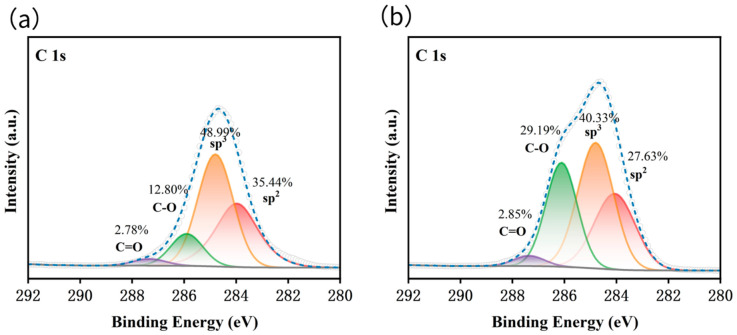
(**a**) XPS spectrum of DND; (**b**) XPS spectrum of the purified product obtained by calcining the DND + KHCO_3_ sample at 350 °C for 2 h. The white circles represent the raw experimental data, and the blue dashed line is the fitted envelope. The orange, red, green, and purple peaks correspond to sp^3^-hybridized carbon, sp^2^-hybridized carbon, C–O bonds, and C=O bonds, respectively. The gray line denotes the background baseline. The relative atomic percentages of each carbon chemical state are labeled next to the corresponding peaks.

To quantitatively analyze changes in the surface chemical composition and carbon hybridization state of DND before and after purification, X-ray photoelectron spectroscopy (XPS) characterization was performed on the original DND black powder and on samples purified by the KHCO_3_ wet wrapping method followed by calcination at 350 °C for 2 h. The high-resolution C 1s spectra and peak fitting results are shown in Figure 5.

As shown in Figure 5a, the C 1s spectrum of the pristine DND can be fitted to four characteristic peaks, corresponding to the sp^2^-hybridized C=C bond (non-diamond carbon impurity) at 284.4 eV, the sp^3^-hybridized C–C bond (intrinsic diamond carbon), the C–O bond at 286.6 eV (oxygen-containing functional groups such as hydroxyl and ether bonds), and the C=O bond at 288.4 eV (carbonyl and lactone groups). The relative content of sp^2^ carbon in the original sample was as high as 35.44%, indicating that its surface was coated with a large amount of non-diamond carbon impurities, which is consistent with the intrinsic structural characteristics of detonation-synthesized DND.

Following purification via low-temperature calcination after wet wrapping with KHCO_3_, the surface carbon species composition of the sample underwent significant changes (Figure 5b). Notably, the relative content of sp^2^ C=C carbon—which represents non-diamond impurities—decreased significantly from 35.44% to 27.63%. It is worth noting that the calcination temperature used in this study was only 350 °C, far below the critical temperature of 400–430 °C required for conventional oxidation in pure air. The effective removal of sp^2^ carbon under such mild reaction conditions directly demonstrates the excellent catalytic activity of alkali metal salts in the oxidation of non-diamond carbon and validates the core advantage of this method: “low-temperature and high-efficiency”.

At the same time, the relative content of oxygen-containing C–O functional groups on the sample surface increased significantly from 12.80% to 29.19%. This is because, after the sp^2^ carbon impurities were oxidized and removed, the fresh diamond crystal surface was exposed and underwent mild oxidation in an air atmosphere at 350 °C, resulting in the formation of a large number of oxygen-containing functional groups such as hydroxyl groups and ether bonds. This change is also fully consistent with the results of the zeta potential measurements described earlier, which showed that “the surface charge of the purified sample shifted from positive to negative,” further confirming the evolution of the surface chemical structure.

#### 3.4.2. Transmission Electron Microscopy (TEM) Analysis

**Figure 6:**
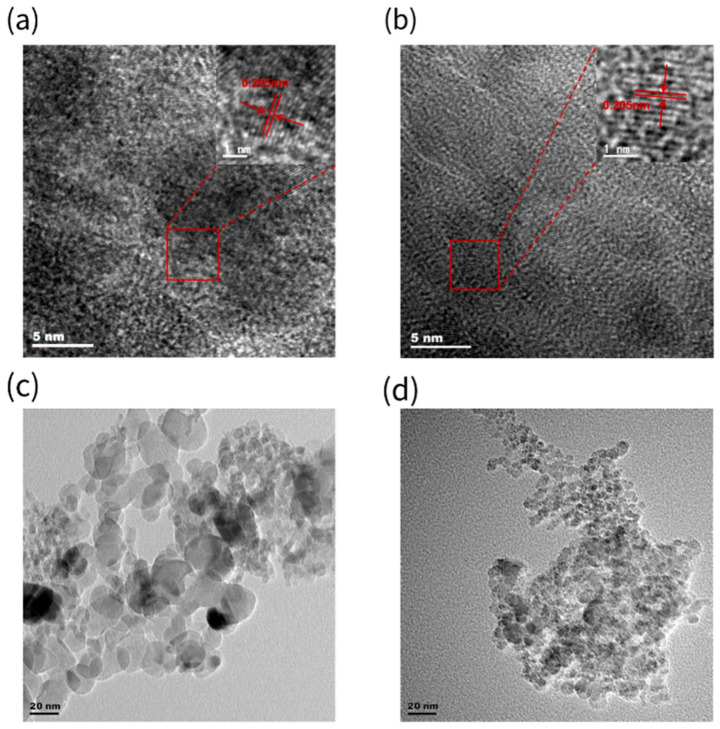
(**a**) DND and (**b**) KHCO_3_-catalyzed purified DND high-resolution HR-TEM images (scale bar: 5 nm), clearly showing diamond (111) lattice striations (0.2055 nm). (**c**) Low-magnification TEM image of DND after purification using (**d**) KHCO_3_ catalyst (scale bar: 20 nm), demonstrating particle dispersion and size distribution.

To verify the stability of nanodiamond structures during salt-assisted low-temperature calcination, transmission electron microscopy (TEM) analysis was performed on the original black powder and on samples obtained by mixing KHCO_3_ with the black powder, calcining at 300 °C for 2 h, and subsequently removing the salt. The results are shown in Figure 6.

In high-resolution TEM images, lattice fringes with an interplanar spacing of approximately 0.205 nm are clearly observable in both sets of samples (Figure 5a,b), corresponding to diamond’s {111} plane [37,38,39,40], indicating that nanodiamond grains were already present in the original black powder. Furthermore, after salt-assisted calcination at 300 °C, their crystal structure remained intact without any destruction or phase transformation. Furthermore, no other new crystalline phases with characteristic lattice spacings were detected within the observed field of view, indicating that the low-temperature calcination process did not introduce new crystalline phases or byproducts.

In TEM images at a larger scale (~20 nm) (Figure 6c,d), the particle size of the purified sample showed a slight overall reduction compared to the original black powder, though the difference was not significant. This result indicates that under relatively mild low-temperature conditions, salt-assisted calcination primarily targets the removal of non-diamond carbon phases, while having limited effects on the size and crystal morphology of nanodiamonds themselves.

Based on thermogravimetric analysis results, it can be concluded that salt-assisted calcination significantly reduces the oxidation temperature of non-diamond carbon while effectively preserving the crystal structure of nanodiamonds. This demonstrates that this low-temperature purification strategy exhibits excellent selectivity and structural retention.

#### 3.4.3. X-Ray Diffraction Analysis

X-ray diffraction (XRD) was employed to analyze the phase composition and crystalline characteristics of the samples before and after salt-assisted low-temperature calcination. The diffraction patterns are shown in Figure 6. Both the DND and the sample calcined at 300 °C with KHCO_3_ assistance and subsequently desalinated (DND + KHCO_3_) exhibited typical diamond diffraction characteristics, indicating that the low-temperature purification process did not cause crystal phase destruction or transformation.

**Figure 7:**
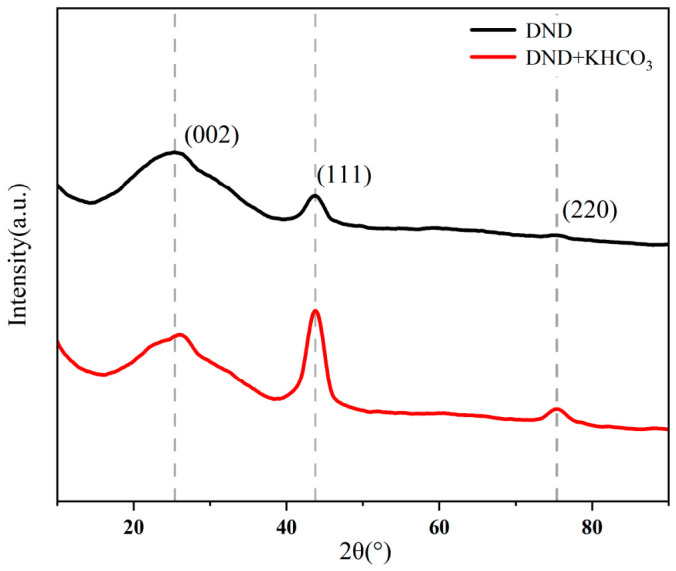
XRD patterns of DND (black line) and DND calcined with KHCO_3_ (red line).

As shown in Figure 7, both sets of samples exhibit distinct diffraction peaks at 2θ ≈ 43.9°, corresponding to the (111) crystal plane of diamond. Additionally, a weak (220) diffraction peak is observed near 2θ ≈ 75.3°, whose position aligns with that of the standard diamond crystal structure [25,39]. These results indicate that after low-temperature calcination treatment assisted by KHCO_3_, the crystal structure of nanodiamonds remains fully preserved without any phase transformation or lattice damage. In the low-angle region (2θ ≈ 25°), both sets of samples exhibit broad diffuse diffraction peaks, typically attributed to the (002) diffraction features of amorphous or disordered carbon phases [38,41]. Compared to the original DND, the relative intensity of this broad peak has diminished in the low-temperature purified sample, while the diffraction characteristics of the diamond (111) peak have become more distinct. This indicates that under salt-assisted calcination conditions, non-diamond carbon phases are preferentially removed, thereby increasing the relative proportion of diamond phase in the sample.

By performing peak shape fitting on the diamond (111) diffraction peaks and calculating the crystal size using the Scherrer formula, the diamond grain sizes of the DND and post-catalytic oxidation samples were determined to be approximately 4.2 nm and 3.1 nm, respectively. Compared to the original DND, the diffraction peaks of the catalyzed oxidation samples exhibited significant broadening, with a corresponding substantial reduction in crystal size. This indicates that the treatment process further diminished the regions of long-range ordered lattice structures within the diamond nuclei. This result indicates that during the purification process, the diamond nuclei underwent a certain degree of structural rearrangement or surface lattice disruption, yet the main diamond phase remained largely intact.

Combining the aforementioned TEM and thermogravimetric analysis results, it can be concluded that KHCO_3_-assisted low-temperature calcination reduces purification temperatures while preserving the crystal integrity and structural stability of nanodiamonds. This further validates the effectiveness of the low-temperature salt-assisted purification strategy at the levels of crystal phase composition and crystallization behavior.

### 3.5. Discussion of Mechanisms

The thermogravimetric analysis (TGA) and transmission electron microscopy (TEM) results in this study jointly demonstrate that various salts exhibit significant catalytic effects on the purification process of detonation-derived nanodiamonds (DNDs). The activity trend is as follows: bicarbonate > carbonate > chloride; Cs^+^ ≈ K^+^ > Na^+^. Building upon the unified mechanism for soot combustion proposed in the pioneering work of Li et al. [42], we can establish a clear and in-depth reaction mechanism model for this catalytic effect.

#### 3.5.1. The Nature of the Active Site: Free Alkali Metal Cations (M^+^)

Li et al. [42] demonstrated through a series of rigorous experiments that the active sites catalyzing soot combustion on potassium-supported oxides are free K^+^ ions, rather than carbonate precursors as previously widely believed. This conclusion provides a key perspective for understanding the catalytic behavior of different salts. Within this research framework, despite employing different salt precursors, they likely converge under reaction conditions, ultimately leading to the same active center: a free alkali metal cation (M^+^).

Specifically, chloride salts (such as NaCl, KCl, CsCl) melt at high temperatures, allowing them to dissociate directly and provide free M^+^ ions. In molten states, carbonate ions (e.g., Na_2_CO_3_, K_2_CO_3_, Cs_2_CO_3_) significantly enhance the electron transfer capacity and catalytic efficiency of M^+^ sites through interface interactions with sp^2^-hybridized carbon atoms. However, their functionality remains fundamentally dependent on the M^+^ core. Bicarbonates (such as NaHCO_3_, KHCO_3_) are first converted into carbonate intermediates, which then function through the same pathway. The wet wrapping process employed essentially creates an optimal microenvironment for full contact between M^+^ active sites and carbon impurities.

Therefore, the catalytic phenomena observed in this study are unified by the core role of free M^+^ ions. The roles of different precursors can be summarized as follows: chlorides serve as direct donors of M^+^ ions, carbonates optimize the active microenvironment for M^+^ through decomposition, while bicarbonates maximize the accessibility and reaction efficiency of M^+^ sites through pretreatment and decomposition pathways. The ultimate activity trend K^+^/Cs^+^ > Na^+^ is determined by the intrinsic physicochemical properties of M^+^ itself (such as ionic radius, charge density, and mobility).

#### 3.5.2. Reaction Pathway: From Carbon Structure Disruption to Unified Intermediate

The catalytic process initiates with the attack of active M^+^ species on the non-diamond carbon structure. According to research by Zhu et al. [43], potassium species diffuse into aromatic rings at high temperatures, forming intercalation compounds (C_n_M). This disrupts the ordered carbon structure, leading to disorder and generating a large number of active carbon sites. This structural rearrangement process establishes the reaction foundation for catalytic oxidation within this system.

In this research system, non-diamond carbon surrounding nanodiamond carbon is activated by metal ions M^+^ to form carbon active sites [44]. Li et al. [42] confirmed through in situ IR and DFT calculations that the ketene (H_2_C=C=O) is a key intermediate in the potassium-catalyzed carbon soot combustion process. We speculate that this intermediate is also present in the DND purification process described in this study. The formation pathway may proceed as follows: activated oxygen species attack the edges or defect sites of the carbon network already activated by M^+^, first forming various oxygen-containing functional groups. Subsequently, through intramolecular rearrangement or further oxidation, highly reactive ketoene (C=C=O) intermediates are formed. The C=C bond in this intermediate is far more fragile than the C–C bond in graphitized carbon, making it highly susceptible to further oxidation into the final product CO_2_.

#### 3.5.3. Catalytic Cycle Mechanism: Synergy of Oxygen Activation, Electron Transfer, and Intermediate Oxidation

**Figure 8:**
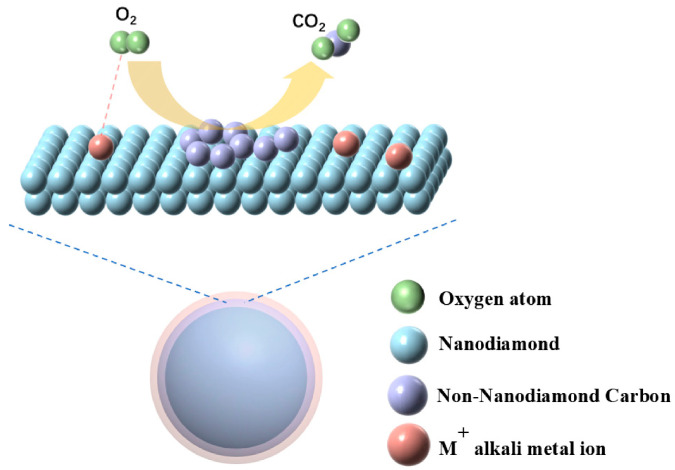
Schematic Model of the Reaction.

Based on the above analysis, we propose a catalytic cycle mechanism applicable to this system, which integrates three key processes: oxygen activation, electron transfer, and intermediate oxidation. Figure 8 depicts a schematic model of the reaction.

Step 1: Oxygen Activation and Electron Transfer: The free M^+^ active site first acts upon the carbon substrate. Previous studies have demonstrated that alkali metal ions can interact significantly with the π electron system of sp^2^-hybridized carbon. This interaction induces electron redistribution or partial transfer from the carbon framework to the M^+^ sites, thereby altering the local electronic structure of the carbon surface. This process particularly enhances the reactivity of edge and defect sites [44,45,46]. This electron transfer process renders the carbon surface, particularly at edge positions, in an electron-rich state. Simultaneously, the M^+^ active site significantly promotes the adsorption and electron transfer of gaseous O_2_ molecules, stabilizing the generation of reduced reactive oxygen species (ROS) such as superoxide (O_2_^−^). This enables effective oxygen activation at lower temperatures [47,48,49].

Step 2: Oxygen Spillover and Intermediate Formation: Reactive oxygen species migrate (spill over) from the M^+^ site to adjacent, activated carbon surfaces, attacking C–C bonds weakened by M^+^ [50]. In this process, ketene is formed as a key unified reaction intermediate.

Step 3: Intermediate Oxidation & Regeneration: The ketene intermediate is rapidly oxidized by reactive oxygen species to form the final products CO and CO_2_, which are then desorbed. The M^+^ site is released, restarting the catalytic cycle. Furthermore, in the bicarbonate system, H_2_O generated in situ during thermal decomposition may further promote the final oxidation of intermediates by forming surface oxygen-containing functional groups such as –OH, thereby synergistically lowering the local reaction energy barrier [24,44].

In summary, the salt-assisted catalytic effect observed in this study can be attributed to a complex synergistic process driven by alkali metal cations (M^+^), involving carbon structure disruption, electron transfer, oxygen activation, and the formation and transformation of ketene intermediates. The differences in activity among various salts fundamentally reflect their varying capacities to provide highly dispersed, highly active M^+^ species, as well as their differing efficiencies in the initial activation of non-diamond carbon structures.

#### 3.5.4. Analysis of the Mechanism Underlying the Superiority of Carbonates

Based on the combined results of thermogravimetric analysis, whiteness measurement, and microstructural characterization, bicarbonate salts (using KHCO_3_ as an example) demonstrate significantly higher catalytic efficiency than chloride and carbonate salts during the purification of nanodiamonds. Its outstanding performance does not stem from a single factor, but rather from the synergistic interaction of three elements: the pretreatment method, the decomposition behavior, and the catalytic microenvironment.

First, the unique solution-coated pretreatment process establishes the optimal initial reaction interface. Unlike the physical mixing employed for chlorides and carbonates, bicarbonates are complexed with DND through a “solution coating method” involving dissolution–evaporation. This process enables bicarbonate crystals to crystallize in situ on the surface and within the pores of DND aggregates, achieving extremely close contact between the salt and carbon reactants at the submicron and even nanoscale [39]. This establishes a highly uniform composite system prior to heat treatment, maximizing the effective contact area for subsequent catalytic reactions.

Second, the multi-step gas–solid reactions during decomposition exert both in situ activation and physical exfoliation effects on the carbon layer. Bicarbonate decomposes at relatively low temperatures (~100–200 °C) (2KHCO_3_ → K_2_CO_3_ + H_2_O↑ + CO_2_↑) [51]. The H_2_O and CO_2_ gases generated during this process rapidly escape within the confined nanoscale space, exerting significant physical fragmentation and perforation effects on the dense sp^2^ carbon layer enveloping the diamond core [52]. This not only compromises the integrity of the carbon layer, exposing numerous new reaction interfaces and defect sites, but also creates pathways for subsequent oxygen and molten salt infiltration. Additionally, the generated H_2_O and CO_2_ themselves can act as weak oxidizing agents (C + H_2_O → CO + H_2_; C + CO_2_ → 2CO), initiating the initial gasification reaction at lower temperatures and further weakening the carbon layer structure [36].

Ultimately, the highly active potassium carbonate generated through conversion inherits and enhances the interfacial catalytic effect. The K_2_CO_3_ formed in situ after bicarbonate decomposition inherits the ideal microscopic contact structure established in the first step and immediately melts at the reaction temperature, initiating the catalytic cycle described in Section 3.5.3.

## 4. Discussion

This study developed and validated a novel strategy for low-temperature air purification using salt-assisted catalytic detonation of nanodiamonds (DND). By mixing DND with salts such as chlorides, carbonates, and bicarbonates and subjecting the mixture to heat treatment in an air atmosphere, the method achieved highly selective removal of sp^2^ non-diamond carbon impurities. Systematic experiments have shown that all tested salts significantly lower the oxidation decomposition temperature of sp^2^ carbon, and their catalytic activity follows a clear pattern: at the anion level, bicarbonates exhibit superior catalytic performance to carbonates, and carbonates, in turn, outperform chlorides. For the same anion, cesium salts and potassium salts exhibit significantly higher catalytic activity than sodium salts. This trend highlights the key role of anions in regulating the reaction microenvironment, as well as the inherent advantages of large-radius, low-electronegativity alkali metal cations in activating oxygen and promoting electron transfer. Unlike the traditional dry-mixing method, the wet-wrapping process not only significantly enhances catalytic efficiency by increasing the contact interface between the salt and DND, but also effectively suppresses particle sintering and agglomeration during high-temperature calcination, thereby greatly improving the aqueous dispersibility of the purified DND. Most importantly, this study is the first to draw upon and integrate classical theories from the field of catalytic combustion of carbonaceous smoke, thereby proposing a unified mechanistic explanation for the salt-assisted DND purification process. It clarifies that free alkali metal cations are the true catalytic active centers, which trigger the reaction through the synergistic action of oxygen release and electron transfer, with ketones and alkenes participating in the catalytic cycle as key intermediates. This mechanism provides a rational explanation for all experimental phenomena observed in this study and offers a theoretical framework for catalyst design in the field of precision processing of carbon materials.

Despite the progress achieved in this study, certain limitations remain to be addressed due to the current experimental conditions and research cycle. The proposed ketene intermediate pathway in the reaction mechanism is currently a reasonable inference based on classical conclusions in the field of soot catalytic combustion and the experimental phenomena of this study, and direct experimental evidence has not yet been obtained through in situ characterization techniques such as in situ infrared spectroscopy and in situ mass spectrometry. Meanwhile, the present work is limited to the laboratory bench scale, and the mass transfer, heat transfer and uniformity issues that may arise during process scale-up have not been thoroughly explored. Subsequent research will be carried out step by step around the above deficiencies: firstly, an in situ thermogravimetry-infrared spectroscopy (TG-IR) platform will be built to detect intermediate species during the reaction in real time, so as to further verify and improve the catalytic reaction mechanism; meanwhile, in situ thermogravimetry-mass spectrometry (TG-MS) and isotope labeling techniques will be adopted to accurately determine the oxidation kinetic parameters of sp^3^ diamond and sp^2^ non-diamond carbon at different temperatures, clarify the oxidation selectivity difference between the two, and provide a theoretical basis for further optimization of process parameters; on this basis, kilogram-scale process scale-up studies will be promoted to optimize industrial reaction parameters and equipment design and solve engineering problems in the scale-up process; finally, the application of this salt-assisted catalytic oxidation strategy in the purification and surface modification of other carbon materials such as carbon nanotubes and graphene will be explored to further expand its scope of application and application value.

## Figures and Tables

**Table 1:** Oxidation temperature data for DND and its different salt mixtures with varying blending ratios. Both ∆T_onset_ and ∆T_peak_ represent the temperature difference relative to the DND group.

Sample	Mixing Method	T_onset_ (°C)	∆T_onset_ (°C)	T_peak_ (°C)	∆T_peak_ (°C)
DND	\	537.5	\	587.2	\
DND + NaCl	Dry-mixing	507.3	−30.2	533.8	−53.4
DND + KCl	Dry-mixing	497.1	−40.4	517.1	−70.1
DND + CsCl	Dry-mixing	479.5	−58.0	509.5	−77.7
DND + Na_2_CO_3_	Dry-mixing	435.8	−101.7	461.3	−125.9
DND + K_2_CO_3_	Dry-mixing	397.9	−139.6	444.2	−143.0
DND + Cs_2_CO_3_	Dry-mixing	386.0	−151.5	453.3	−133.9
DND + Na_2_CO_3_	Wet-wrapping	424.7	−112.8	452.1	−135.1
DND + K_2_CO_3_	Wet-wrapping	395.1	−142.4	435.4	−151.8
DND + NaHCO_3_	Wet-wrapping	411.2	−126.3	438.5	−148.7
DND + KHCO_3_	Wet-wrapping	377.8	−159.7	423.8	−163.4

**Table 2:** Whiteness values and product yields of DND treated with different salt systems and mixing methods.

Sample	Mixing Method	Calcination Temperature-Time	Whiteness (%)	Yield (%)
DND	\	\	0.8 ± 0.04	\
DND	\	425 °C—2 h	1.2 ± 0.06	92.8 ± 1.7
DND	\	350 °C—2 h	0.9 ± 0.03	98.7 ± 0.5
DND + NaCl	Dry-mixing	350 °C—2 h	1.0 ± 0.06	96.5 ± 2.7
DND + KCl	Dry-mixing	350 °C—2 h	1.2 ± 0.03	95.8 ± 1.8
DND + CsCl	Dry-mixing	350 °C—2 h	1.7 ± 0.05	93.5 ± 2.3
DND + Na_2_CO_3_	Dry-mixing	350 °C—2 h	1.6 ± 0.09	93.1 ± 1.2
DND + K_2_CO_3_	Dry-mixing	350 °C—2 h	1.7 ± 0.03	90.5 ± 4.8
DND + Cs_2_CO_3_	Dry-mixing	350 °C—2 h	1.8 ± 0.02	90.7 ± 2.4
DND + Na_2_CO_3_	Wet-wrapping	350 °C—2 h	3.5 ± 0.08	86.7 ± 1.5
DND + K_2_CO_3_	Wet-wrapping	350 °C—2 h	13.6 ± 0.13	84.6 ± 3.8
DND + NaHCO_3_	Wet-wrapping	350 °C—2 h	3.7 ± 0.03	88.7 ± 2.0
DND + KHCO_3_	Wet-wrapping	350 °C—2 h	14.4 ± 0.11	83.8 ± 3.6

**Table 3:** Particle size, polydispersity index and zeta potential of different treatment groups.

Group	Z-Ave (nm)	Intensty Mean (nm)	Volume Mean (nm)	Number Mean (nm)	PDI	Zeta (mV)
DND group	724.67 ± 8.56	604.8 ± 9.8	518.77 ± 37.71	156.17 ± 3.14	0.57 ± 0.05	37.26 ± 0.76
DND + Na_2_CO_3_ Comparison	243.73 ± 3.37	346.5 ± 37.88	348.93 ± 310.79	96.24 ± 30.68	0.23 ± 0.02	17.99 ± 0.94
DND + Na_2_CO_3_ Dry-mixing	438.7 ± 10.5	292.17 ± 14.27	223.9 ± 102.65	193.47 ± 116.63	0.39 ± 0.05	−18.92 ± 1.51
DND + Na_2_CO_3_ Wet-wrapping	199 ± 5.29	281.9 ± 23.23	208.73 ± 124.33	80.97 ± 14.6	0.23 ± 0	−24.16 ± 1.17
DND + K_2_CO_3_ Comparison	2591.33 ± 336.3	1747.33 ± 1054.54	1870 ± 1208.04	1587.67 ± 904.52	0.45 ± 0.16	−20.35 ± 1.48
DND + K_2_CO_3_ Dry-mixing	384.9 ± 9	362.7 ± 19.61	304.73 ± 112.91	159.7 ± 146.81	0.32 ± 0.03	22.15 ± 1.38
DND + K_2_CO_3_ Wet-wrapping	234.87 ± 5.92	318.7 ± 19.68	142.83 ± 16.66	66.21 ± 10.78	0.25 ± 0.04	28.00 ± 1.05

## Data Availability

The original contributions presented in this study are included in the article. Further inquiries can be directed to the corresponding author.
